# Association of echocardiographic findings with mortality: human assessment vs. automated deep learning analysis

**DOI:** 10.1093/ehjdh/ztaf148

**Published:** 2026-02-03

**Authors:** Roei Merin, Moran Gvili Perelman, Hila Merin, Maor Tzuberi, Shmuel Banai, Elina Stsiapanava, Yan Topilsky, Nir Flint

**Affiliations:** Internal Medicine Division, Tel Aviv Sourasky Medical Center, Tel Aviv, Israel; Department of Cardiology, affiliated to the Tel Aviv University School of Medicine, 6 Weizmann st, Tel Aviv 64239, Israel; Ben Gurion University of the Negev, School of Medicine, Beer Sheva, Israel; Faculty of Medicine,Tel Aviv University, Tel Aviv, Israel; Department of Cardiology, affiliated to the Tel Aviv University School of Medicine, 6 Weizmann st, Tel Aviv 64239, Israel; Department of Cardiology, affiliated to the Tel Aviv University School of Medicine, 6 Weizmann st, Tel Aviv 64239, Israel; Department of Cardiology, affiliated to the Tel Aviv University School of Medicine, 6 Weizmann st, Tel Aviv 64239, Israel; Department of Cardiology, affiliated to the Tel Aviv University School of Medicine, 6 Weizmann st, Tel Aviv 64239, Israel

**Keywords:** Echocardiography, Artificial Intelligence, Deep Learning, Strain Imaging

## Abstract

**Aims:**

Artificial intelligence (AI) has emerged as a promising tool for echocardiographic image analysis, potentially improving efficiency and reducing inter-observer variability. Real-world comparisons between AI-based analysis and human expert interpretation, and their correlation to clinical outcomes, remain limited. This study aimed to evaluate the correlation between AI-based echocardiographic analysis and human expert interpretation and to compare their association with one-year mortality in hospitalized patients.

**Methods and results:**

We conducted a retrospective analysis of 889 consecutive hospitalized patients who underwent a clinically indicated echocardiographic exam. All studies were read and analysed by both human echocardiographic experts and by commercially available AI software (Us2.ai). We performed correlation analysis of common echocardiographic variables obtained by human vs. AI and compared their performance in the prediction of 1-year mortality. Of the 889 patients, 731 (82%) patients (mean age 68 ± 16, 46% Females) had sufficient echocardiographic data to be included in the analysis. Most parameters exhibited a strong correlation between human and AI-derived measurements. AI-derived LVEF values were significantly higher than human estimates (mean difference 5.8%, *P* < 0.001). In a multivariable model, AI- and human-based mortality prediction were comparable (AUC 0.67 vs. 0.66, *P* = 0.86). When including AI-obtained automated left ventricular strain analysis, the AI-based model was superior to humans in predicting 1-year mortality (AUC 0.73 vs. 0.66, *P* = 0.048).

**Conclusion:**

AI-based echocardiographic analysis shows excellent correlation with human-derived measurements. Incorporating automated strain analysis resulted stronger association with mortality of the AI analysis compared to standard human analysis.

## Graphical Abstract

**Graphical Abstract:**
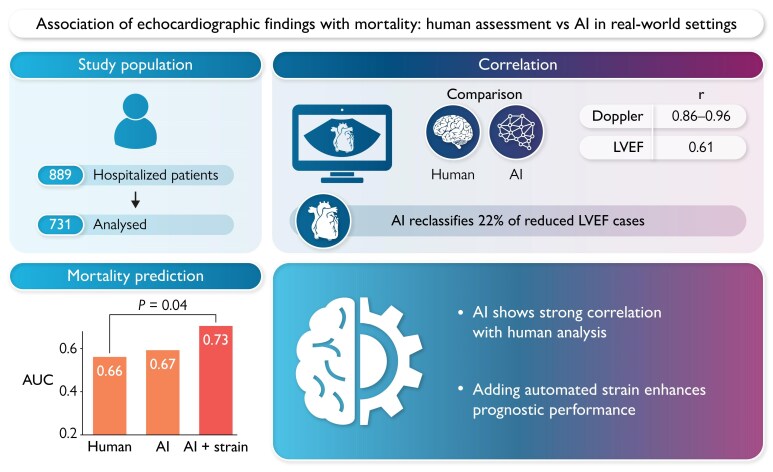

## Background

The use of artificial intelligence (AI) in echocardiography has been rapidly advancing for both image acquisition and image analysis.^1–3^ AI algorithms can identify echocardiographic images^4,5^ and perform automatic measurements and analysis. Several validation studies have shown that AI-based analysis has good accuracy for left ventricular function assessment,^6–8^ allowing reduced operator variability and improved efficiency compared to standard human analysis. AI algorithms can also perform automated analysis and interpretation of echocardiographic strain imaging, with comparable accuracy to conventional measurements.^9^

Fully automated AI analysis has the ability to change routine workflow and potentially reduce growing workloads. Several AI-based software are commercially available, but real-world data on the integration of such applications in routine clinical use is limited. *Us2.ai* is a commercially available deep-learning algorithm for automated echocardiographic analysis, previously validated for automated left ventricular volumes and function measurements, strain measurements, and valve dysfunction.^9–11^

Standard echocardiographic parameters, such as left ventricular ejection fraction (LVEF), diastolic function, and pulmonary pressures, are associated with clinical outcomes and mortality.^12–14^ It is unclear whether echocardiographic parameters obtained by AI algorithms have a comparable ability to predict clinical outcomes.

This study aims to perform a real-world comparison between AI-based and human expert-based echocardiographic measurements and the association of these parameters with all-cause mortality in hospitalized patients.

## Methods

### Data collection

This is a single-centre, historic, prospective study. We analysed consecutive echocardiographic examinations conducted during January 2020 at the Tel-Aviv Medical Center, a tertiary hospital with a high-volume echocardiography lab. The study was reviewed and approved by the Institutional Review Board in compliance with the ethical standards outlined in the Declaration of Helsinki. The need for informed consent was waived by the ethical committee. We screened all hospitalized patients who underwent a clinically indicated comprehensive transthoracic echocardiography (TTE) exam over a period of one month, regardless of the reason for admission or for the echocardiographic exam. TTE studies were performed by trained sonographers using the E95 systems (GE Vingmed Ultrasound, Horten, Norway) or the iE33/Epiq systems (Philips Medical Systems, Andover, MA). Clinical data were obtained from electronic medical records. Mortality data were obtained from the national death registry database.

### Image Analysis

All TTE studies were routinely interpreted and analysed by human echocardiographic experts, followed by an automated AI-based analysis. Human-based echocardiographic measurements were obtained in accordance with the guidelines of the American Society of Echocardiography, except for left ventricular ejection fraction (LVEF), which was visually estimated (‘eye-balling’) in 5% increments. The AI analysis was performed by a machine-learning-based algorithm (Us2.AI, Singapore), which was previously validated.^9,15^ This software is commercially available and has been cleared by the US Food and Drug Administration. LVEF was calculated by the AI algorithm using the biplane Simpson’s method.

No manual adjustments or interventions were made on the automated AI-based analysis, and human readers were blinded to the AI-derived measurements. Studies with inadequate image quality that prevented a complete analysis by the AI algorithm were excluded, specifically when more than 20% of the selected echocardiographic parameters were missing (shown in *Table 1*).

**Table 1:** Clinical characteristics of the studied population

Clinical characteristic	Value
Age, years (mean ± SD)	68 ± 17
Females (*n*, %)	335 (46%)
Ischemic heart disease (*n*, %)	190 (26%)
COPD (*n*, %)	69 (9%)
Diabetes mellitus (*n*, %)	230 (31%)
Congestive heart failure (*n*, %)	212 (29%)
Atrial fibrillation (*n*, %)	173 (24%)
Hyperlipidemia (*n*, %)	321 (44%)
Cigarette smoking (*n*, %)	67 (9%)

COPD, chronic obstructive pulmonary disease.

### Statistical analysis

The primary endpoint of the study was one-year all-cause mortality. All data were analysed using SPSS version 21.0. Continuous variables are described as mean ± standard deviation (SD) and categorical variables as value (%). *P* value <0.05 was considered statistically significant.

We analysed the correlation between standard echocardiographic parameters measured by human experts to those measured by the AI algorithm. We focused on 12 primary echo parameters of left ventricular (LV) systolic and diastolic function and right ventricular (RV) function. For variables with normal distribution, correlation was assessed using a *T*-test for the difference of mean, otherwise a Mann–Whitney analysis was conducted. Correlation coefficients were calculated using Pearson correlation for parameters with normal distribution and Spearman correlation for parameters not normally distributed or non-linear (i.e. LVEF). We used Bland-Altman plots to graphically describe the agreement between measurements.

To predict 1-year all-cause mortality, we constructed multivariable logistic regression models separately for human-derived and AI-derived parameters. Variables were selected for multivariable analysis based on statistical significance (*P* < 0.05) in univariable analysis, followed by forward stepwise selection. Parameters that improved model performance were retained. Cut-off values were identified using a decision tree and the Chi-squared Automatic Interaction Detector (CHAID) algorithm. Each parameter was analysed as continuous or binary (above or below the cut-off) according to its significance in mortality prediction. At each step, we compared models based on their ability to predict mortality using the area under the curve (AUC) of the Receiver Operator Characteristic (ROC) curve. To ensure validity, we also assessed multicollinearity using variance inflation factors (VIF), with a cut-off of 5. Differences between AUCs were compared using the DeLong test.

To assess human variability, a second blinded cardiologist independently re-measured LVEF in a random sample of 50 studies. Inter-observer agreement was calculated using a two-way random-effects intraclass correlation coefficient for absolute agreement of single measurements [ICC(2,1)] with 95% CI.

## Results

Of the 889 patients, 731 (82%) patients (mean age 68 ± 16, 46% Females) were included in the analysis. Reasons for exclusion were incomplete AI analysis (73 patients, mainly due to poor acoustic window), incomplete human echo report (25 patients), missing clinical data (25 patients), and other reasons (35 patients), as software and technical errors during the AI analysis. *Table 2* shows the demographic and clinical data of the included patients. The excluded patients did not differ in their demographic and clinical characteristics compared with the included patients.

**Table 2:** Correlation of echocardiographic measurements obtained by human vs. AI

Measurement	Human	AI	R correlation	ICC (3,1) [95% CI]	Mean bias	Limits of agreement	
						lower	upper
LV Diastolic Diameter, mm (mean, ±SD)	47.3 ± 7	44.6 ± 6.9	0.78	0.78 (0.75–0.81)	2.91	−5.91	11.72
LV Systolic Diameter, mm (mean ± SD)	31.2 ± 8.1	31.1 ± 7.8	0.81	0.81 (0.78–0.83)	0.40	−9.27	10.08
LV posterior wall thickness, mm (mean ± SD)	9.7 ± 1.7	9.3 ± 1.9	0.52	0.52 (0.46–0.58)	0.41	−3.16	3.98
LV Septum Thickness, mm (mean ± SD)	10.8 ± 2.2	10.2 ± 2.1	0.65	0.65 (0.6–0.69)	0.67	−2.90	4.23
LVOT VTI, cm (mean ± SD)	20.1 ± 5.0	21.8 ± 5.6	0.88	0.87 (0.85–0.88)	−1.61	−6.96	3.75
LVEF, % (median, IQR)	60 (50,60)	63 (53,69)	0.61	0.7 (0.67–0.74)	−5.73	−22.19	10.73
LV peak E velocity, cm/s (median, IQR)	75.4 (60,93)	80 (64,97)	0.96	0.96 (0.95–0.97)	−3.97	−17.6	9.7
E/e’ (median, IQR)	12 (9,17)	10 (8,14)	0.86	0.85 (0.83–0.88)	2.10	−3.77	7.96
RV TAPSE, mm (mean, ±SD)	2.07 ± 0.52	2.03 ± 0.46	0.69	0.68 (0.64–0.73)	0.54	−7.08	8.15
RV Peak S Velocity, cm/s (mean, ±SD)	11.4 ± 3.1	12 ± 3.2	0.84	0.84 (0.81- 0.86)	−1.4	−11.3	8.5
RA area, cm^2^ (median, IQR)	15 (13,19)	13 (11,16)	0.77	0.84 (0.81–0.86)	2.43	−3.56	8.42
SPAP, mmHg (median, IQR)	32 (27,42)	30 (24,38)	0.88	0.88 (0.86–0.9)	1.45	−11.03	13.93

LV, left ventricular; LVOT VTI, left ventricular outflow tract velocity time integral; LVEF, left ventricular ejection fraction; RV, right ventricular; TAPSE, tricuspid annular plane systolic excursion; RA, right atrium; SPAP, systolic pulmonary arterial pressure.

**Figure 1:**
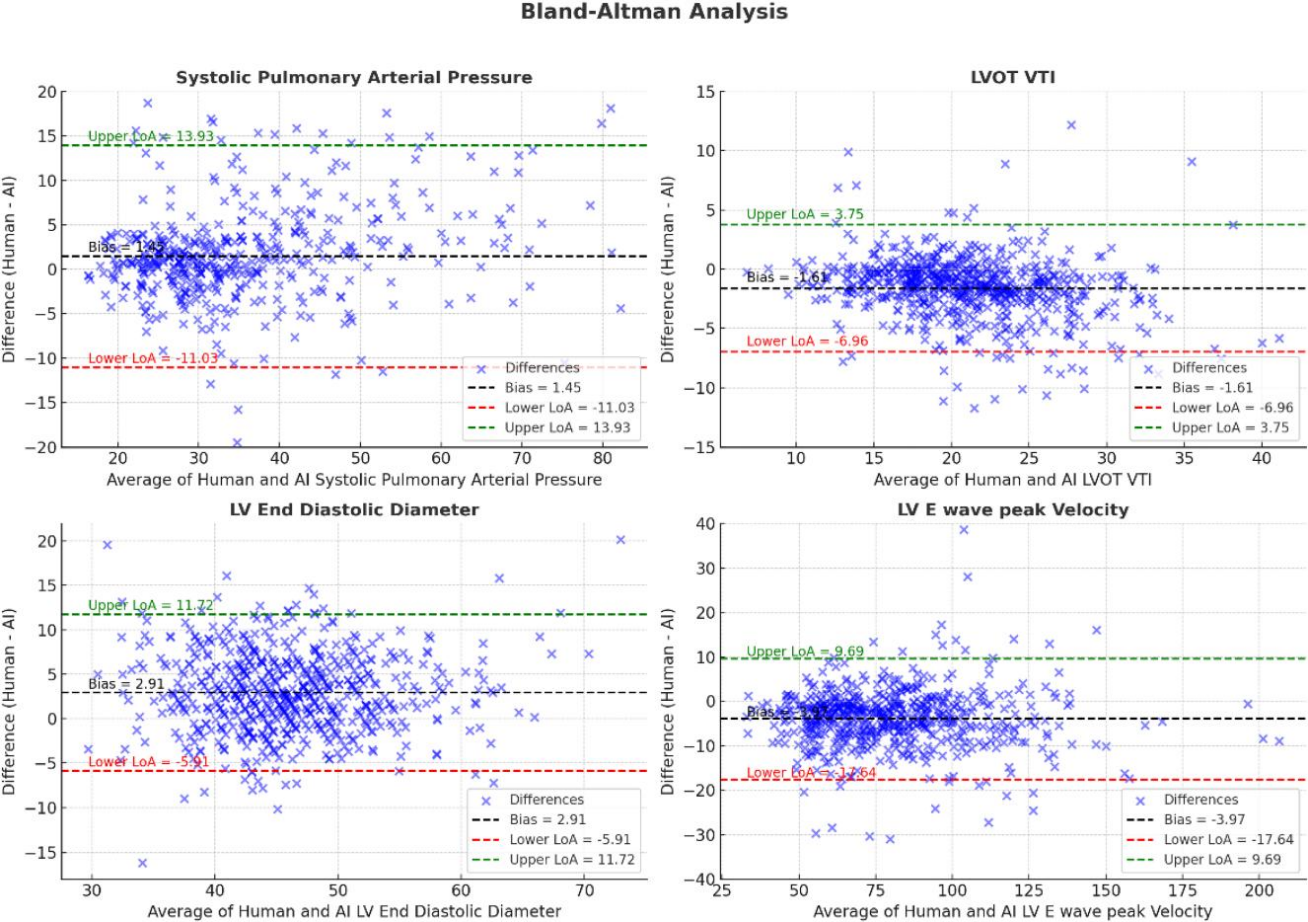
Bland–Altman plots for correlation between human vs. AI-based analysis.

**Figure 2:**
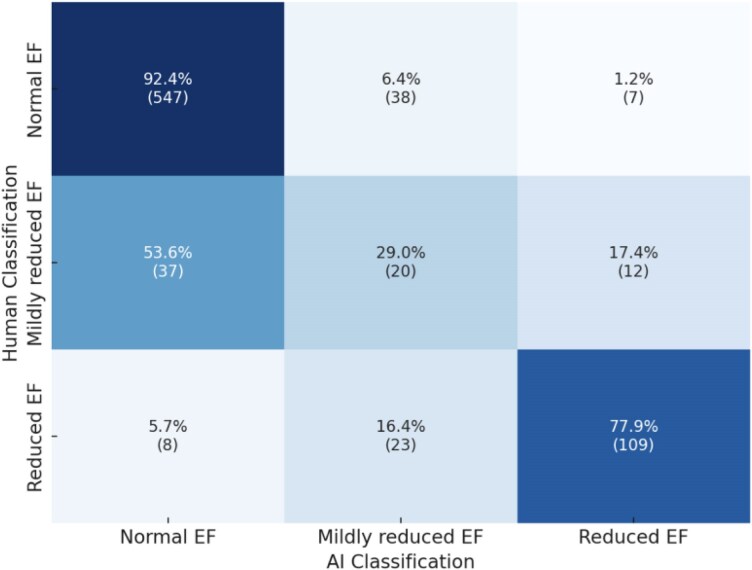
Re-classification of EF class using the AI algorithm (normal LVEF ≥ 50%, mildly reduced 50%>LVEF ≥ 40%, and reduced LVEF < 40%).

*Table 1* shows the correlations between human- and AI-obtained measurements, and Bland-Altaman Analysis are shown in *Figure 1*. Parameters with the strongest correlation were E wave velocity (*r*  *=* 0.96, *P* < 0.001), E/e’ ratio (*r*  *=* 0.86, *P* < 0.001), and systolic pulmonary artery pressure (*r*  *=* 0.88, *P* < 0.001). LV wall thickness showed moderate correlation between human and AI measurements; however, with an absolute mean difference that is not clinically significant. Left ventricular ejection fraction (LVEF) also showed moderate correlation (*r*  *=* 0.61), with AI-obtained LVEF values being significantly higher compared to human-estimated LVEF values (mean difference of 5.8, *P* < 0.001). The resulting re-classification between LVEF categories (normal LVEF ≥ 50%, mildly reduced 50%>LVEF ≥ 40%, reduced LVEF < 40%) is shown in *Figure 2*.

The inter-observer agreement between the two human readers was good, with an intraclass correlation coefficient [ICC(2,1)] of **0.716** (95% CI **0.778–0.918**), indicating a high level of consistency between human measurements.

Ninety-five (13%) patients died during the first year after the index TTE, and overall mortality was 24% during a follow-up period of 53 months. The predictive power for 1-year mortality of each echocardiographic parameter obtained by human or AI is presented in *Table 3*. In univariable analysis, 15 parameters automatically obtained by AI were significantly predictive of 1-year mortality compared with 9 parameters from the human-based analysis.

**Table 3:** Human- and AI-obtained echocardiographic parameters: univariable analysis for 1-year all-cause mortality

		Odds ratio	95% CI		*P* value	AUC	AUC difference	*P* value
LV diameter-diastolic	AI	0.99	0.96	1.02	0.4	0.60	0.08	0.22
	Human	1.01	0.97	1.03	0.8	0.52		
LV diameter-systolic	AI	1.01	0.99	1.04	0.37	0.54	−0.01	0.48
	Human	1.01	0.99	1.04	0.78	0.55		
Septum thickness	AI	1.19	1.08	1.32	<0.001	0.62	−0.01	0.64
	Human	1.2	1.09	1.32	<0.001	0.62		
Posterior wall thickness	AI	1.27	1.12	1.43	<0.001	0.65	0.03	0.48
	Human	1.27	1.12	1.44	<0.001	0.62		
EF	AI	0.97	0.96	0.99	<0.001	0.61	0.03	0.29
	Human	0.97	0.96	0.99	0.01	0.58		
LVOT-VTI	AI	0.95	0.91	0.99	0.01	0.57	−0.01	0.51
	Human	0.94	0.9	0.98	0.01	0.58		
Peak E velocity	AI	1.01	1.01	1.02	<0.001	0.63	0.02	0.88
	Human	1.01	1.01	1.02	<0.001	0.61		
E/e'	AI	1.06	1.02	1.1	<0.001	0.57	−0.06	0.04
	Human	1.07	1.03	1.11	<0.001	0.63		
RVs’	AI	0.9	0.83	0.97	0.01	0.59	0.01	0.54
	Human	0.91	0.85	0.98	0.02	0.58		
TAPSE	AI	0.92	0.88	0.98	0.01	0.58	0.03	0.4
	Human	0.97	0.93	1.02	0.23	0.55		
SPAP	AI	1.04	1.03	1.06	<0.001	0.68	−0.01	0.56
	Human	1.05	1.03	1.07	<0.001	0.69		
RA area	AI	1.1	1.05	1.14	<0.001	0.62	0.01	0.76
	Human	1.07	1.03	1.11	0.01	0.61		
LV-GLS	AI	0.89	0.83	0.94	<0.001	0.65		
LA reservoir strain	AI	0.98	0.96	1	0.02	0.59		

AI, artificial intelligence; AUC, area under the curve; LV, left ventricle; EF, ejection fraction; LVOT-VTI, left ventricular outflow tract - velocity time integral; E/e', early diastolic mitral inflow velocity to tissue Doppler velocity ratio; RVs', right ventricular systolic velocity; TAPSE, tricuspid annular plane systolic excursion; SPAP, systolic pulmonary artery pressure; RA, right atrium; GLS, global longitudinal strain.

**Figure 3:**
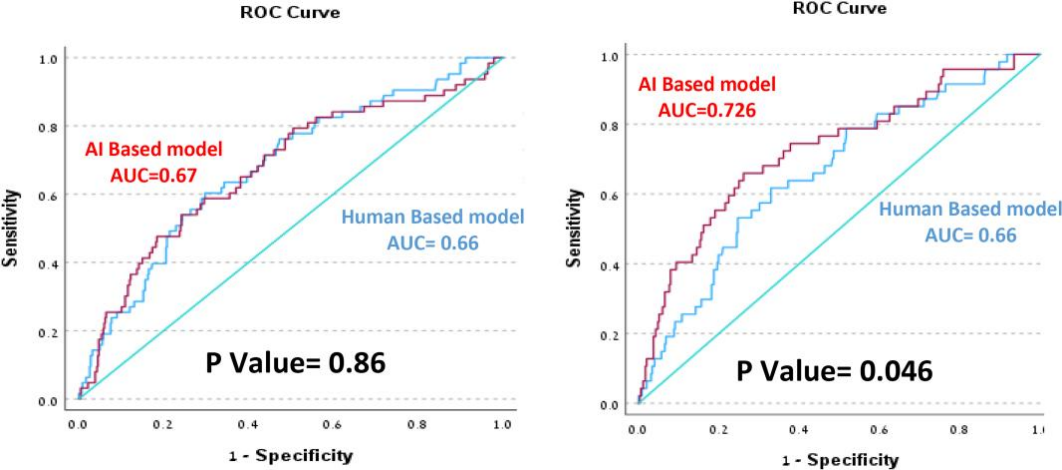
Comparative ROC analysis for AI vs. human-assessed echocardiographic measurements in predicting 1-year mortality. (*A*) shows human vs. AI model 1, and (*B*) human vs. AI model 2.

Echocardiographic parameters that were predictive of 1-year mortality in the univariable analysis (*P* < 0.05) were considered for inclusion in the multivariable model. Using the forward stepwise selection approach, we identified three key echocardiographic parameters that significantly improved model performance: Posterior wall thickness, mitral E wave velocity, and LVEF ≤45%. These variables were used to construct logistic regression models based on either human-derived or AI-derived measurements (*Table 4a*). The predictive performance of the two models was comparable, with no statistically significant difference (AUC 0.66 vs. 0.67, *P* = 0.86), as shown in *Figure 3*. We then constructed a second AI-based model that included additional parameters routinely measured only by the AI software – specifically, strain measurements. This model demonstrated improved prognostic performance for predicting 1-year mortality compared to the human-based model (AUC 0.73 vs. 0.66, *P* = 0.048; *Table 4b*).

**Table 4a:** Human-based model with comparable AI model (AI model 1)

	Huma-based model		AI-based model	
	Odds ratio (95% CI)	*P* Value	Odds ratio (95% CI)	*P* Value
Posterior wall thickness (mm)	1.21 (1.05–1.38)	0.006	1.21 (1.06–1.36)	0.003
Peak E wave velocity (cm/s)	1.01 (1.003–1.020)	0.011	1.01 (1.05–1.1)	0.001
LVEF ≤ 45%	2.11 (1.21–3.69)	<0.001	1.244 (0.6–2.6)	0.562

**Table 4b:** AI model for 1-year mortality prediction including strain parameters (AI model 2)

	Odds ratio (95% CI)	*P* value
Posterior wall thickness >9.5 mm	2.65 (1.41–4.98)	0.002
Mitral E velocity	1.01(1.003–1.025)	0.010
LV GLS ≤18%	2.47 (1.28–4.76)	0.007
Left atrial reservoir strain	1.01 (0.98–1.04)	0.48

## Discussion

Our study aimed to compare echocardiographic measurements obtained by a fully automated, deep learning AI-based algorithm to those of human expert readers in a real-world setting. The main findings of this study were (1) the majority of echocardiographic parameters demonstrated good or excellent correlation between human- and machine-based measurements. (2) LVEF values obtained by AI showed moderate correlation with human-based values and were overall higher than human-estimated LVEF, and (3) in a multivariable model for 1-year mortality, AI-based measurements showed stronger association with 1-year mortality compared with human expert analysis, attributed to the incorporation of strain measurements in all AI-based analysis.

In echocardiography, human expert interpretation is considered the gold standard for detecting various cardiac conditions, such as systolic and diastolic function and the degree and mechanism of valvular pathologies. Deep learning AI applications provide automated measurements and interpretation of standard echocardiographic images and Doppler signals. Compared to human-based interpretation, AI-based analysis has potential advantages: (1) automatic border detection for volumetric analysis (e.g. Simpson's method) and speckle-tracking strain analysis, (2) averaging Doppler tracings over multiple cardiac cycles, and (3) inclusion of measurements not routinely performed in standard transthoracic echocardiograms protocols, such as speckle-tracking strain of the left ventricle, right ventricle, and atria. These additional capabilities enhance the completeness of the echocardiographic report and may provide incremental prognostic information.

The heterogeneous nature of echocardiographic images, their operator and reader dependency, and variability in image quality pose challenges in any platform intended for automatic echocardiography analysis. This was evident in our study, as only 82% of studies had adequate image quality to allow the AI platform to perform a complete analysis.

For most linear measurements, such as LV dimensions and wall thickness, we observed a strong correlation between AI-obtained and human-expert measurements. Our findings are consistent with previous reports showing a good correlation between AI and human experts for standard echocardiographic measurements. In a previous study by Tromp *et al.*^15^ the investigators validated deep learning-based automatic echocardiographic analysis for systolic and diastolic function​. Similar to our study, Doppler parameters obtained by AI showed good correlation to human measurements. For example, we found a correlation coefficient of *r*  *=* 0.86 for E/e’ ratio, compared to *r*  *=* 0.75–0.9 in the study by Tromp. Comparable correlation was also found for LV dimensions (*r*  *=* 0.81 compared to *r*  *=* 0.83–0.9).

It is common practice in many echocardiography labs to perform a visual estimation (‘eye-balling’) of the LVEF. When performed by experienced clinicians, visual estimation correlates well with biplane Simpson’s technique and three-dimensional echocardiography.^16–18^ However, visual estimation tends to systematically underestimate LVEF- a trend also observed in our study, where human-derived LVEF was visually assessed, while AI-derived LVEF was calculated using automated biplane Simpson’s. Human readers often integrate knowledge of the patient's condition – such as recent myocardial infarction or suspected regional wall motion abnormalities – into their assessment. This can lead to a lower reported LVEF, even when accurate volumetric measurements (e.g. Simpson’s or 3D echocardiography) indicate a normal value. While this approach may enhance clinical relevance in certain scenarios, it introduces subjectivity and inter-operator variability. This raises an important question: what is the *clinically relevant* LVEF – the one perceived and interpreted in the context of the patient’s presentation, or the one derived through objective, standardized quantification? According to our results, in patients initially classified as having normal LVEF, 7.5% were classified by the AI as having reduced or mildly reduced LVEF. 54% of patients initially classified as mildly reduced were classified as normal LVEF by the AI analysis, and 22% of patients initially classified with reduced LVEF(<40%) were analysed by the AI as having mildly reduced or normal LVEF. With current guidelines for congestive heart failure treatment, this potential re-classification may change patient management and prognostication.

Of note, other studies, using different techniques for LVEF evaluation, reported better correlation between human and AI assessments, with *r* values ranging from 0.75 to 0.89.^15^ In a cohort of patients with COVID-19, AI-based LVEF and strain analysis were superior to humans in predicting mortality and demonstrated lower inter-operator variability.^19^ In another study, AI was superior to sonographers for LVEF assessment, showing less variability and better consistency with cardiologist interpretations.^6^

We aimed to assess the ability of automated deep learning analysis to predict clinical outcomes, as compared to human analysis. Comparing the two methods using similar echocardiographic parameters in the models for the prediction of 1-year mortality, resulted in similar AUC (0.66 and 0.67, *P* = 0.86). This implies that the differences in the 2D and Doppler measurements observed between the two methods may not be clinically significant.

Routine utilization of speckle-tracking echocardiography for left ventricular (LV), right ventricular (RV), and left atrial (LA) strain assessment requires technical expertise and can be time-consuming. While semi-automated and fully automated platforms have improved the usability and reproducibility of strain analysis, it is still not routinely performed in all standard TTE studies. While the clinical importance of routine strain analysis is well documented, practical barriers – primarily related to time, cost, and expertise – limit widespread integration, making AI-based solutions a compelling alternative.

Adding myocardial strain analysis to the model, which is automatically performed in all AI-analysed TTEs (but is not routinely performed in human-based analysis), yielded a higher AUC for predicting 1-year mortality (0.726 vs. 0.66; *P* = 0.046). This suggests that while AI-derived standard measurements are comparable to those of human analysis in predicting mortality, the more comprehensive nature of the AI analysis – including automated strain – may enhance the clinical and prognostic utility of echocardiographic evaluation.

## Limitations

This study has several limitations. We excluded 18% of the original cohort due to suboptimal image quality and other technical issues that did not allow for complete AI analysis. In addition, this is a heterogeneous cohort as we included consecutive patients admitted for different conditions, including surgical patients. Thus, for some patients, the cause of death may be non-cardiac and unrelated to the indication for which a TTE was ordered.

## Conclusion

AI-based echocardiographic analysis shows excellent correlation with human-derived measurements. The incorporation of automated strain analysis – not routinely available in human interpretation – was associated with a stronger prediction of mortality, highlighting the potential prognostic value of comprehensive automated AI-based analysis.

## Data Availability

The data underlying this article will be shared on reasonable request to the corresponding author.
